# SCANNER: a web platform for annotation, visualization and sharing of single cell RNA-seq data

**DOI:** 10.1093/database/baab086

**Published:** 2022-01-17

**Authors:** Guoshuai Cai, Xuanxuan Yu, Choonhan Youn, Jun Zhou, Feifei Xiao

**Affiliations:** Department of Environmental Health Sciences, Arnold School of Public health, University of South Carolina, Columbia, SC 29208, USA; Department of Epidemiology and Biostatistics, Arnold School of Public Health, University of South Carolina, Columbia, SC 29208, USA; San Diego Supercomputer Center, University of California San Diego, La Jolla, CA 92093, USA; Research Computing Group, University of South Carolina, Columbia, SC 29208, USA; Department of Epidemiology and Biostatistics, Arnold School of Public Health, University of South Carolina, Columbia, SC 29208, USA

## Abstract

In recent years, efficient scRNA-seq methods have been developed, enabling the transcriptome profiling of single cells massively in parallel. Meanwhile, its high dimensionality and complexity bring challenges to the data analysis and require extensive collaborations between biologists and bioinformaticians and/or biostatisticians. The communication between these two units demands a platform for easy data sharing and exploration. Here we developed Single-Cell Transcriptomics Annotated Viewer (SCANNER), as a public web resource for the scientific community, for sharing and analyzing scRNA-seq data in a collaborative manner. It is easy-to-use without requiring special software or extensive coding skills. Moreover, it equipped a real-time database for secure data management and enables an efficient investigation of the activation of gene sets on a single-cell basis. Currently, SCANNER hosts a database of 19 types of cancers and COVID-19, as well as healthy samples from lungs of smokers and non-smokers, human brain cells and peripheral blood mononuclear cells (PBMC). The database will be frequently updated with datasets from new studies. Using SCANNER, we identified a larger proportion of cancer-associated fibroblasts cells and more active fibroblast growth-related genes in melanoma tissues in female patients compared to male patients. Moreover, we found *ACE2* is mainly expressed in lung pneumocytes, secretory cells and ciliated cells and differentially expressed in lungs of smokers and never smokers.

**Key Points**

- Single-Cell Transcriptomics Annotated Viewer (SCANNER) provides a new web resource to bridge the data analysis and the biological experiment units.
- SCANNER enables comprehensive and dynamic analysis, visualization, functional annotation and easy data sharing.
- SCANNER hosts a real-time database for data and user management.

## Introduction

In recent years, single-cell RNA sequencing (scRNA-seq) methods have been enabling the fast transcriptome profiling of individual cells (1). It provides opportunities to identify cell clusters with their specific biomarkers and insights into cell developmental trajectory, gene bursting activity, cell interaction and others (2). However, the high dimensionality and complex properties (e.g. zero-inflation, overdispersion, batch effect) of scRNA-seq data brought challenges in its analysis. A widely used strategy is to reduce the high dimension into an embedding space of two- or three-dimensions, and in which further data analysis such as clustering could be performed. Generally, scRNA-seq data analysis asks for multiple-step data processing, including quality control, read mapping, count matrix construction, normalization, dimension reduction, clustering, downstream analyses, visualization and interpretation. These processes require extensive knowledge of data modeling and computational skills, as well as experiment design, study goals and biological insights. Therefore, a close collaboration between bioinformaticians and biologists is greatly needed in scRNA-seq studies. However, tools for assisting that collaboration by easy data sharing and exploration are missing. To bridge the data analysis and the biological experiment units, we developed the Single-Cell Transcriptomics Annotated Viewer (SCANNER), as a new web platform for scRNA-seq data management, sharing, analysis and interpretation in a comprehensive, flexible and collaborative manner.

## Results

**Figure 1.**
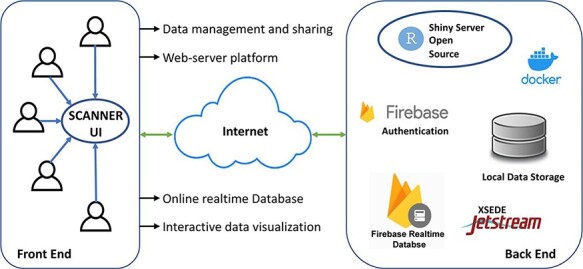
The SCANNER architecture. SCANNER facilitates the sharing, visualization, analysis and interpretation of scRNA-seq data and the communication between biologists and bioinformaticians in a flexible and collaborative manner.

SCANNER was constructed using R 4.2.0 and R shiny package and is currently built on a CentOS Linux 7 server, with 6 CPUs and 16GB memory in the environment of Jetstream (3, 4). The data in SCANNER is securely managed by Google Firebase (https://firebase.google.com). SCANNER enables a unique set of functions highlighted in Figure 1. Specifically

- SCANNER enables the user data management and sharing easily, without hosting the authentication and database infrastructure locally. Users can generate SCANNER data objects by following the instruction in **Availability and implementation** (see below **SCANNER data object generation**) and uploading them to SCANNER. For secure data management, a user account is required, and the registration is broadly open to the scientific community. After users logged into the SCANNER, they can upload/delete their own datasets and share/unshare them with collaborators who have registered in SCANNER. When users share datasets with specific collaborators, SCANNER sends an email about the file-sharing information to each of those user-designated collaborators. Also, users can access publicly pre-processed datasets hosted in SCANNER, which is described below.
- SCANNER is a web server application. Several local applications such as single-cell viewer (SCV) (5), Cerebro (6) and others (7) have been developed for scRNA-seq data interactive visualization. However, they require local installation and data pre-processing which could still set obstacles to biologists for efficient data exploration. As a web server application, SCANNER requires no software setup and enables host-free work, fulfilling the demand of efficient communication for seamless collaboration. Different existing web applications [e.g. the Broad Institute Single Cell Portal (https://portals.broadinstitute.org/single_cell) and scRNASeqDB (8)] are mainly for publicly available datasets analysis. SCANNER allows users to manage, share and analyze their own datasets. Root in SCV (5), SCANNER provides five visualization modules for identified clusters (Cluster), gene expression in cells (Expression), distribution of gene expression in clusters (Distribution), expression detection rate and size in clusters (Detection) and expression heatmap with hierarchical clustering across genes and cells (Similarity). Each module dynamically takes parameters set by users for feature selection, group comparison, cluster filtering and layout setting.
- SCANNER provides an online database for scRNA-seq data. Due to the rapid development of new technologies, the volume and complexity of data increase quickly and highly require an effective gateway for data exploration. Databases such as the Broad Institute Single Cell Portal (https://portals.broadinstitute.org/single_cell) and scRNASeqDB (8) are available but their implemented functions are static and limited for data visualization and analysis. Currently, SCANNER hosts a database of 50 datasets from 19 types of cancers, lungs of smokers and non-smokers, airways and blood of covid-19 patients and healthy controls, healthy human brain cells and peripheral blood mononuclear cells (PBMC). We will continue to collect datasets and frequently update SCANNER to provide a comprehensive resource of single-cell transcriptomics for public use.
- SCANNER provides functional annotation and activeness inference. Gene set analysis can provide valuable insights into explaining biological mechanisms in terms of biologically relevant sets, such as signaling pathways. To enable this, SCANNER infers and visualizes the activation status of a particular gene set that is involved in a gene set by four scores (**Supplementary Methods**), including average expression, average expression rank, eigen-gene expression (9) and enrichment score (10).

Here, we demonstrate the application of SCANNER with two case studies:

- **Sex disparity in melanoma-associated fibroblast.** We analyzed the melanoma dataset (11) and found that tumours from female patients had significantly larger proportions of cancer-associated fibroblasts (CAF) and endothelial cells than those from males (Supplementary Figure S1A). Correspondingly, in CAF and endothelial cells of the female patients, the fibroblast growth factor (FGF) binding function inferred by enrichment score is highly activated (Fig. S1B, C) with higher detection rates and activeness scores (Supplementary Figure S1D), which were confirmed by methods of average expression, average rank and eigen-gene expression (Supplementary Figure S2). Consistently, the FGF genes FGF1 and FGF2 are highly expressed in CAF cells in the melanoma tissue from female patients (Supplementary Figure S3). Such over-expression was also found in most of the genes involved in the FGF binding function (Supplementary Figure S4). Given that CAF is a promising target to treat melanoma (12), this gender difference in CAF may link to the observation that male patients usually have worse survival outcomes compared to females (13).
- **Tobacco-use disparity in *ACE2* lung expression.** Exploring our database of the smoking lung, we found that the gene of the SARS-CoV-2 receptor, ACE2, is mainly expressed in pneumocytes, secretory cells and ciliated cells (Supplementary Figure S5), which is consistent with the recent study of Ziegler *et al.* (14). Also, among bronchial epithelial cells, we found that the ACE2 gene is mainly expressed in club cells in never smokers. Differently in smokers, goblet cells are extensively proliferated and harbour most expressed ACE2, which may indicate a complex effect of smoking on the COVID-19 risk (15).

### SCANNER data object generation

SCANNER requires scRNA-seq data preprocessing from the user-end, using a Seurat preprocessing workflow which has been provided in https://github.com/GuoshuaiCai/scanner. Within the Seurat framework, the data preprocessing workflow including data normalization, high variable feature selection, data scaling, dimension reduction and cluster identification can be performed with the user-defined parameters according to the data, experiment design and knowledge. As demonstrated in the preprocessing workflow, SCANNER also requires meta information, phenotype information (if applicable) and the top 10, 30 and 100 differentially expression genes. Currently, SCANNER focused on the efficient exploration of gene expression but is incapable of visualizing other inferences such as RNA velocity and psuedotime, which is considered a limitation of SCANNER. However, SCANNER is compatible with Seurat objects generated from the user’s analysis pipeline by automatically extracting information from necessary data slots.

## Discussion

In this study, we developed SCANNER for supporting efficient data sharing and communication between biologists and bioinformaticians, which is expected to promote scRNA-seq studies in the scientific community broadly. In future updates, SCANNER will be equipped with the capacity to host and manage single-cell spatial transcriptomics and multimodal omics data, and is expected to be compatible with other data processing workflows, such as scanpy (16) and scater (17).

## Supplementary Material

baab086_Supp

## Availability and implementation

SCANNER is available at https://www.thecailab.com/scanner/. Data processing instructions and supporting functions are available at https://github.com/GuoshuaiCai/scanner.
