# Supplementary material for: SCANNER: a web platform for annotation, visualization and sharing of single cell RNA-seq data
Source: Database (Oxford). 2022 Jan 17;2022:baab086. doi: 10.1093/database/baab086 (PMC9246089; doi:10.1093/database/baab086)
Supplement: baab086_Supp [file baab086_supp.zip › additional file 1_R1_clean ...docx]

SCANNER: A Web Platform for Annotation, Visualization and Sharing of Single Cell RNA-seq Data

### Guoshuai Cai^1^, Xuanxuan Yu^2^, Choonhan Youn^3^, Jun Zhou^4^, Feifei Xiao^2^

^1^Department of Environmental Health Sciences, ^2^Department of Epidemiology and Biostatistics, Arnold School of Public Health, University of South Carolina, Columbia, SC 29208, US.

^3^ San Diego Supercomputer Center, University of California, San Diego, La Jolla, CA 92093, US.^4^ Research Computing Group, University of South Carolina, Columbia, SC 29208, US.

**Short title:** A Web Resource for scRNA-seq Data

**Corresponding Authors:**

Guoshuai Cai, PhD

Department of Environmental Health Sciences

Arnold School of Public Health

University of South Carolina
921 Assembly Street

Public Health Research Center 401C

Columbia, SC 29204

GCAI@mailbox.sc.edu

Phone: 803-777-4120

Feifei Xiao, PhD

Department of Epidemiology and Biostatistics

Arnold School of Public Health

University of South Carolina
915 Greene Street, Room 449

Columbia, SC 29204

xiaof@mailbox.sc.edu

Phone: 803-777-8936

**Supplementary Methods**

**Single-cell RNA-seq datasets**

A total of 50 datasets are current publicly available in SCISSOR. All datasets were preprocessed using Seurat 3.0 for data normalization, scaling and dimension reduction. Default parameters were used in all steps if not specified below. In most datasets expect PBMC and COVID-19 datasets, cell types were identified by their original studies or following the same approach used in each study.

*Cancers*

We searched for scRNA-seq data for cancers in PubMed, GEO databases and several other databases. After filtering out data without cell type annotation, we finally obtained publicly available large-scale scRNA-seq datasets for 19 cancer types, including Colon cancer (GSE81861 (1), GSE146771 (2), GSE108989 (3), GSE178341 (4)), Head and neck squamous cell carcinoma (GSE103322 (5)), Breast cancer (GSE114725 (6)), Glioblastoma (GSE84465 (7)), Liver cancer (CNP0000650 (8), GSE140228 (9)), Bile duct cancer (GSE125449 (10)), Cholangiocarcinoma (GSE125449 (10)), Acute myeloid leukemia (GSE116256 (11)), Melanoma (GSE72056 (12), GSE123139 (13)), Lung Adenocarcinoma (GSE131907 (14), E-MTAB-6149 (15)), Pancreatic cancer (GSE154763(16), CRA001160 (17)), Kidney cancer (GSE154763 (16), Young et al. (18)), Lymphoma (heiDATA (19), GSE154763 (16)), Myeloma (GSE154763(16)), Uterine corpus endometrial carcinoma (GSE154763 (16)), Esophageal carcinoma (GSE154763 (16)), Ovarian cancer (GSE154763 (16), GSE158722 (20))), Thyroid carcinoma (GSE154763 (16)) and Squamous cell cancer (GSE144236 (21)).

*Healthy brain and PBMC*

SCANENR also prepressed and hosted two data sets from healthy human samples, including GSE67835 (22) (healthy human brain) and PBMC 3k dataset from 10X Genomics (<https://support.10xgenomics.com/single-cell-gene-expression/datasets/1.1.0/pbmc3k>). The cell types in the PBMC dataset were identified based on the gene expression of known makers, including IL7R and CCR7 for Naïve CD4+ T cells, CD14 and LYZ for CD14+ Monocytes, IL7R and S100A4 for Memory CD4+ T cells, MS4A1 for B cells, CD8A for CD8+ T cells, FCGR3A and MS4A7 for GCGR3A+ Monocytes, GNLY and NKG7 for NK cells, FCER1A and CST3 for DC cells, and PPBP for Platelets.

*Lungs of smokers and non-smokers*

Three publicly available datasets from lung samples of smokers and non-smokers were preprocessed, including the dataset from bronchial epithelial cells, ALCAM+ epithelial cells and CD45+ white blood cells from six never and six current smokers (23), the dataset from lung tissue of one former smoker, two current smoker and five non-smokers) (24) and the dataset from lung tissue of one former smoker, one current smoker and three non-smokers (25).

*Dendritic* *cells in* *upper airway, lower airway and peripheral blood samples from COVID-19 patients and healthy controls*

scRNA-seq datasets of dendritic cells (DCs) in upper airway (nasopharynx/pharynx samples), lower airway (bronchoalveolar lavage fluid (BALF) samples) and peripheral blood (peripheral blood mononuclear cell (PMBC) samples) from COVID-19 patients and healthy controls were processed. The data characteristics and processing were described in our recent published study(26).

**Gene set activity inference**

Four approaches are available in SCANNER for inferring the relative activeness of a particular pathway, function or set of interested genes across cells. In one of all $q$ cells, the activeness score $S$ of an interested gene set, which has $m$ genes, is calculated by,

- *Average expression*, that

$$S=\frac{1}{m}\sum_{i=1}^{m} E_{i}$$

where $E_{i}$ is the scRNA-seq normalized data of the $i$-th gene. We used Seurat normalized read count in *log* scale in developing the smoking lung database.

- *Average rank, that*

$$S=\frac{1}{m}\sum_{i=1}^{m} R_{i}$$

where $R_{i}$ is the rank of the $i$-th gene.

- *Eigen-gene expression,* which can be considered as a weighted average expression from a gene set*.* Briefly, the covariance matrix $C_{m\times m}$ of the gene set expression matrix is calculated by

$$C_{m\times m}=\frac{1}{q-1}X^{T}X$$

where $X$ is the zero centered and unit variance scaled expression matrix of $q\times m$ size, where $q$ is the number of cells and $m$ is the number of genes of interest. Since the covariance matrix $C$ is symmetric and positive definite, the eigenvalues of $C$ are real and it can be diagonalized as

$$C=V\Lambda V^{T}$$

where $V$ is the eigenvector matrix and $\Lambda$ is an eigenvalue matrix. The principal components (PCs) can be obtained by $XV$ and $S$ is given by the first PC (PC1) which represents the most variation in the data. To reduce the effect of extreme values on SCANNER visualization, we constrain $S$ within its 5^th^-95^th^ percentiles.

- *Gene set enrichment score,* which is calculated using a similar strategy of single sample gene set enrichment analysis (ssGSEA) (27)*.* For the gene set $G$ of size $m$ from all $n$ genes in a sample,

$$S=\sum_{j=1}^{n} \left[ \sum_{i\in G,i\leq j} \frac{{E_{i}}^{\alpha}}{\sum_{i\in G} {E_{i}}^{\alpha}}-\sum_{i\notin G,i\leq j} \frac{1}{\left( n-m \right)} \right],$$

where $E_{i}$ gives more weight on highly expressed genes, and $\alpha$ controls the degree of the weight. In this study, SCANNER set $\alpha=1$ which was typically used in the regular ssGSEA.

Current activity inference is available for MSigDB v.7.0 (28) gene sets of Hallmark Collection, KEGG Pathway, Biocarta Pathway, Reacome Pathway, GO Biological Process, GO Cellular Component, GO Molecular Function, Oncogenic Signatures and Immunological Signatures.

**Data downsampling**

SCANNER downsamples data to maintain a favorable computing speed and resource usage. Currently, maximum 500 cells for each cluster are selected by SCANNER using two approaches:

*Random sampling*: cells in each cluster are randomly selected.

*Prediction ellipse*: cells in an fitted ellipse which surround a particular cluster at a quantile of max(1, $\frac{500}{the cell number in cluster})$ are selected. The ellipse can be easily defined by its covariance matrix and vector of means in a low-dimensional space of scRNA-seq data, which is t-distributed Stochastic Neighbor Embedding (t-SNE) (29) embeddings currently used by SCANNER. The axis scales of ellipse were calculated using the square root of the eigenvalues of the covariance matrix and then the distance to ellipse can be solved. R Package “SIBER” was used for realizing this function. This method selects the core cells that are most distinct for each cluster.

Functions for above methods are available at https://github.com/GuoshuaiCai/scanner.

**References**

1. Li, H., Courtois, E.T., Sengupta, D., Tan, Y., Chen, K.H., Goh, J.J.L., Kong, S.L., Chua, C., Hon, L.K., Tan, W.S. *et al.* (2017) Reference component analysis of single-cell transcriptomes elucidates cellular heterogeneity in human colorectal tumors. *Nat Genet*, **49**, 708-718.

2. Zhang, L., Li, Z., Skrzypczynska, K.M., Fang, Q., Zhang, W., O'Brien, S.A., He, Y., Wang, L., Zhang, Q., Kim, A. *et al.* (2020) Single-Cell Analyses Inform Mechanisms of Myeloid-Targeted Therapies in Colon Cancer. *Cell*, **181**, 442-459 e429.

3. Zhang, L., Yu, X., Zheng, L., Zhang, Y., Li, Y., Fang, Q., Gao, R., Kang, B., Zhang, Q., Huang, J.Y. *et al.* (2018) Lineage tracking reveals dynamic relationships of T cells in colorectal cancer. *Nature*, **564**, 268-272.

4. Pelka, K., Hofree, M., Chen, J.H., Sarkizova, S., Pirl, J.D., Jorgji, V., Bejnood, A., Dionne, D., Ge, W.H., Xu, K.H. *et al.* (2021) Spatially organized multicellular immune hubs in human colorectal cancer. *Cell*, **184**, 4734-4752 e4720.

5. Puram, S.V., Tirosh, I., Parikh, A.S., Patel, A.P., Yizhak, K., Gillespie, S., Rodman, C., Luo, C.L., Mroz, E.A., Emerick, K.S. *et al.* (2017) Single-Cell Transcriptomic Analysis of Primary and Metastatic Tumor Ecosystems in Head and Neck Cancer. *Cell*, **171**, 1611-1624 e1624.

6. Azizi, E., Carr, A.J., Plitas, G., Cornish, A.E., Konopacki, C., Prabhakaran, S., Nainys, J., Wu, K., Kiseliovas, V., Setty, M. *et al.* (2018) Single-Cell Map of Diverse Immune Phenotypes in the Breast Tumor Microenvironment. *Cell*, **174**, 1293-1308 e1236.

7. Darmanis, S., Sloan, S.A., Croote, D., Mignardi, M., Chernikova, S., Samghababi, P., Zhang, Y., Neff, N., Kowarsky, M., Caneda, C. *et al.* (2017) Single-Cell RNA-Seq Analysis of Infiltrating Neoplastic Cells at the Migrating Front of Human Glioblastoma. *Cell Rep*, **21**, 1399-1410.

8. Sun, Y., Wu, L., Zhong, Y., Zhou, K., Hou, Y., Wang, Z., Zhang, Z., Xie, J., Wang, C., Chen, D. *et al.* (2021) Single-cell landscape of the ecosystem in early-relapse hepatocellular carcinoma. *Cell*, **184**, 404-421 e416.

9. Zhang, Q., He, Y., Luo, N., Patel, S.J., Han, Y., Gao, R., Modak, M., Carotta, S., Haslinger, C., Kind, D. *et al.* (2019) Landscape and Dynamics of Single Immune Cells in Hepatocellular Carcinoma. *Cell*, **179**, 829-845 e820.

10. Ma, L., Hernandez, M.O., Zhao, Y., Mehta, M., Tran, B., Kelly, M., Rae, Z., Hernandez, J.M., Davis, J.L., Martin, S.P. *et al.* (2019) Tumor Cell Biodiversity Drives Microenvironmental Reprogramming in Liver Cancer. *Cancer Cell*, **36**, 418-430 e416.

11. van Galen, P., Hovestadt, V., Wadsworth Ii, M.H., Hughes, T.K., Griffin, G.K., Battaglia, S., Verga, J.A., Stephansky, J., Pastika, T.J., Lombardi Story, J. *et al.* (2019) Single-Cell RNA-Seq Reveals AML Hierarchies Relevant to Disease Progression and Immunity. *Cell*, **176**, 1265-1281 e1224.

12. Tirosh, I., Izar, B., Prakadan, S.M., Wadsworth, M.H., 2nd, Treacy, D., Trombetta, J.J., Rotem, A., Rodman, C., Lian, C., Murphy, G. *et al.* (2016) Dissecting the multicellular ecosystem of metastatic melanoma by single-cell RNA-seq. *Science*, **352**, 189-196.

13. Li, H., van der Leun, A.M., Yofe, I., Lubling, Y., Gelbard-Solodkin, D., van Akkooi, A.C.J., van den Braber, M., Rozeman, E.A., Haanen, J., Blank, C.U. *et al.* (2019) Dysfunctional CD8 T Cells Form a Proliferative, Dynamically Regulated Compartment within Human Melanoma. *Cell*, **176**, 775-789 e718.

14. Kim, N., Kim, H.K., Lee, K., Hong, Y., Cho, J.H., Choi, J.W., Lee, J.I., Suh, Y.L., Ku, B.M., Eum, H.H. *et al.* (2020) Single-cell RNA sequencing demonstrates the molecular and cellular reprogramming of metastatic lung adenocarcinoma. *Nat Commun*, **11**, 2285.

15. Lambrechts, D., Wauters, E., Boeckx, B., Aibar, S., Nittner, D., Burton, O., Bassez, A., Decaluwe, H., Pircher, A., Van den Eynde, K. *et al.* (2018) Phenotype molding of stromal cells in the lung tumor microenvironment. *Nat Med*, **24**, 1277-1289.

16. Cheng, S., Li, Z., Gao, R., Xing, B., Gao, Y., Yang, Y., Qin, S., Zhang, L., Ouyang, H., Du, P. *et al.* (2021) A pan-cancer single-cell transcriptional atlas of tumor infiltrating myeloid cells. *Cell*, **184**, 792-809 e723.

17. Peng, J., Sun, B.F., Chen, C.Y., Zhou, J.Y., Chen, Y.S., Chen, H., Liu, L., Huang, D., Jiang, J., Cui, G.S. *et al.* (2019) Single-cell RNA-seq highlights intra-tumoral heterogeneity and malignant progression in pancreatic ductal adenocarcinoma. *Cell Res*, **29**, 725-738.

18. Young, M.D., Mitchell, T.J., Vieira Braga, F.A., Tran, M.G.B., Stewart, B.J., Ferdinand, J.R., Collord, G., Botting, R.A., Popescu, D.M., Loudon, K.W. *et al.* (2018) Single-cell transcriptomes from human kidneys reveal the cellular identity of renal tumors. *Science*, **361**, 594-599.

19. Roider, T., Seufert, J., Uvarovskii, A., Frauhammer, F., Bordas, M., Abedpour, N., Stolarczyk, M., Mallm, J.P., Herbst, S.A., Bruch, P.M. *et al.* (2020) Dissecting intratumour heterogeneity of nodal B-cell lymphomas at the transcriptional, genetic and drug-response levels. *Nat Cell Biol*, **22**, 896-906.

20. Nath, A., Cosgrove, P.A., Mirsafian, H., Christie, E.L., Pflieger, L., Copeland, B., Majumdar, S., Cristea, M.C., Han, E.S., Lee, S.J. *et al.* (2021) Evolution of core archetypal phenotypes in progressive high grade serous ovarian cancer. *Nat Commun*, **12**, 3039.

21. Ji, A.L., Rubin, A.J., Thrane, K., Jiang, S., Reynolds, D.L., Meyers, R.M., Guo, M.G., George, B.M., Mollbrink, A., Bergenstrahle, J. *et al.* (2020) Multimodal Analysis of Composition and Spatial Architecture in Human Squamous Cell Carcinoma. *Cell*, **182**, 497-514 e422.

22. Darmanis, S., Sloan, S.A., Zhang, Y., Enge, M., Caneda, C., Shuer, L.M., Hayden Gephart, M.G., Barres, B.A. and Quake, S.R. (2015) A survey of human brain transcriptome diversity at the single cell level. *Proc Natl Acad Sci U S A*, **112**, 7285-7290.

23. Duclos, G.E., Teixeira, V.H., Autissier, P., Gesthalter, Y.B., Reinders-Luinge, M.A., Terrano, R., Dumas, Y.M., Liu, G., Mazzilli, S.A., Brandsma, C.A. *et al.* (2019) Characterizing smoking-induced transcriptional heterogeneity in the human bronchial epithelium at single-cell resolution. *Sci Adv*, **5**, eaaw3413.

24. Reyfman, P.A., Walter, J.M., Joshi, N., Anekalla, K.R., McQuattie-Pimentel, A.C., Chiu, S., Fernandez, R., Akbarpour, M., Chen, C.I., Ren, Z. *et al.* (2019) Single-Cell Transcriptomic Analysis of Human Lung Provides Insights into the Pathobiology of Pulmonary Fibrosis. *Am J Respir Crit Care Med*, **199**, 1517-1536.

25. Madissoon, E., Wilbrey-Clark, A., Miragaia, R.J., Saeb-Parsy, K., Mahbubani, K.T., Georgakopoulos, N., Harding, P., Polanski, K., Huang, N., Nowicki-Osuch, K. *et al.* (2019) scRNA-seq assessment of the human lung, spleen, and esophagus tissue stability after cold preservation. *Genome Biol*, **21**, 1.

26. Cai, G., Du, M., Bosse, Y., Albrecht, H., Qin, F., Luo, X., Androulakis, X.M., Cheng, C., Nagarkatti, M., Nagarkatti, P. *et al.* (2021) SARS-CoV-2 Impairs Dendritic Cells and Regulates DC-SIGN Gene Expression in Tissues. *Int J Mol Sci*, **22**.

27. Barbie, D.A., Tamayo, P., Boehm, J.S., Kim, S.Y., Moody, S.E., Dunn, I.F., Schinzel, A.C., Sandy, P., Meylan, E., Scholl, C. *et al.* (2009) Systematic RNA interference reveals that oncogenic KRAS-driven cancers require TBK1. *Nature*, **462**, 108-112.

28. Liberzon, A., Subramanian, A., Pinchback, R., Thorvaldsdottir, H., Tamayo, P. and Mesirov, J.P. (2011) Molecular signatures database (MSigDB) 3.0. *Bioinformatics*, **27**, 1739-1740.

29. van der Maaten, L. and Hinton, G. (2008) dimensionality_reduction visualization. *Journal of Machine Learning Research*, **9**, 2579-2605.

**Supplementary Figures**


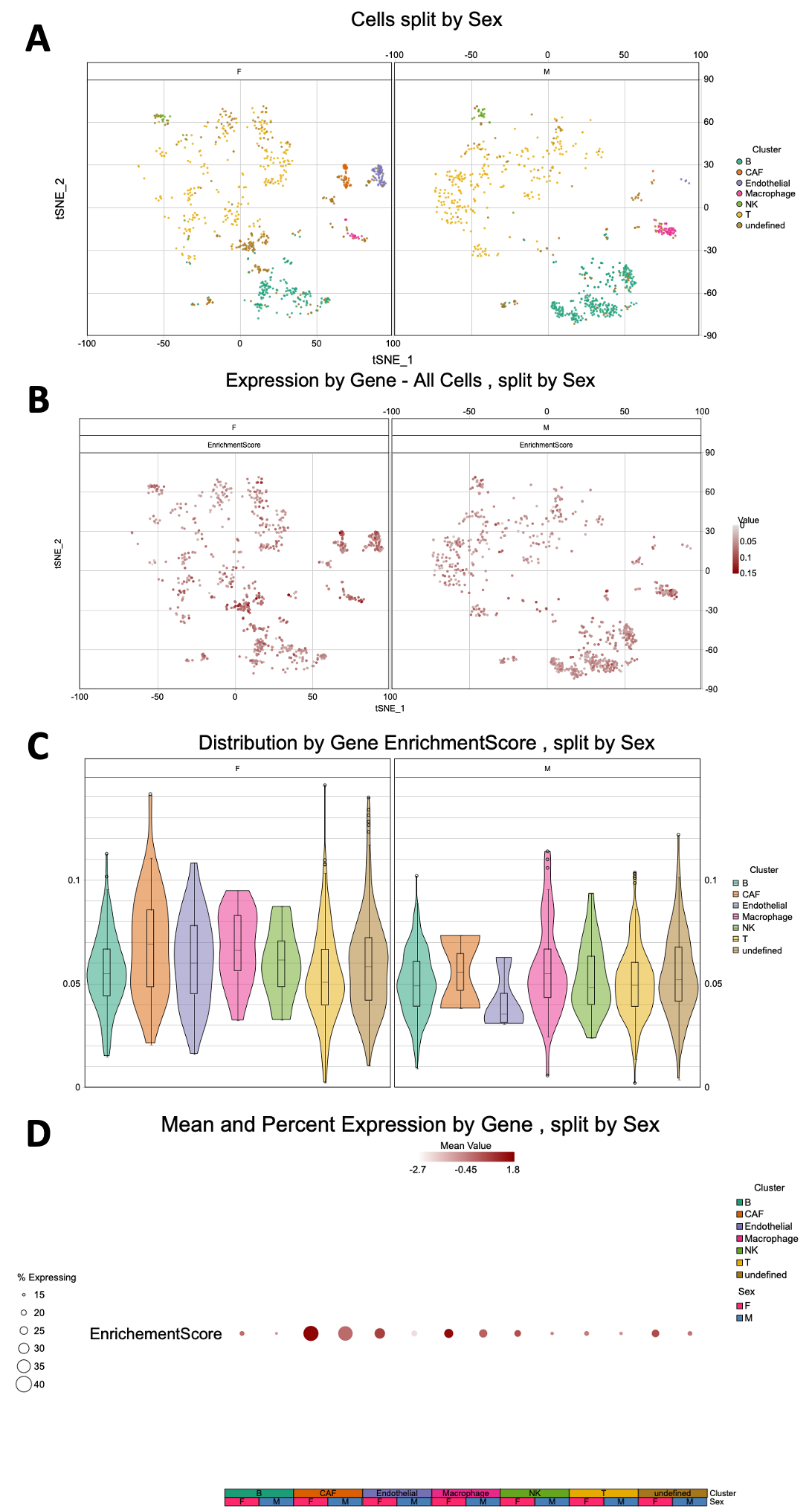


**Figure S1. SCANNER identify sex disparity in melanoma-associated fibroblast.** The disparity is found in (A) cell cluster, (B) enrichment score of GO: fibroblast growth factor binding function, (C) enrichment score distribution and (D) enrichment score level and detection rate. Darker color indicates higher activity in (B) and (D) and a larger size indicates a higher detection rate in (D). F: Female, M: Male.


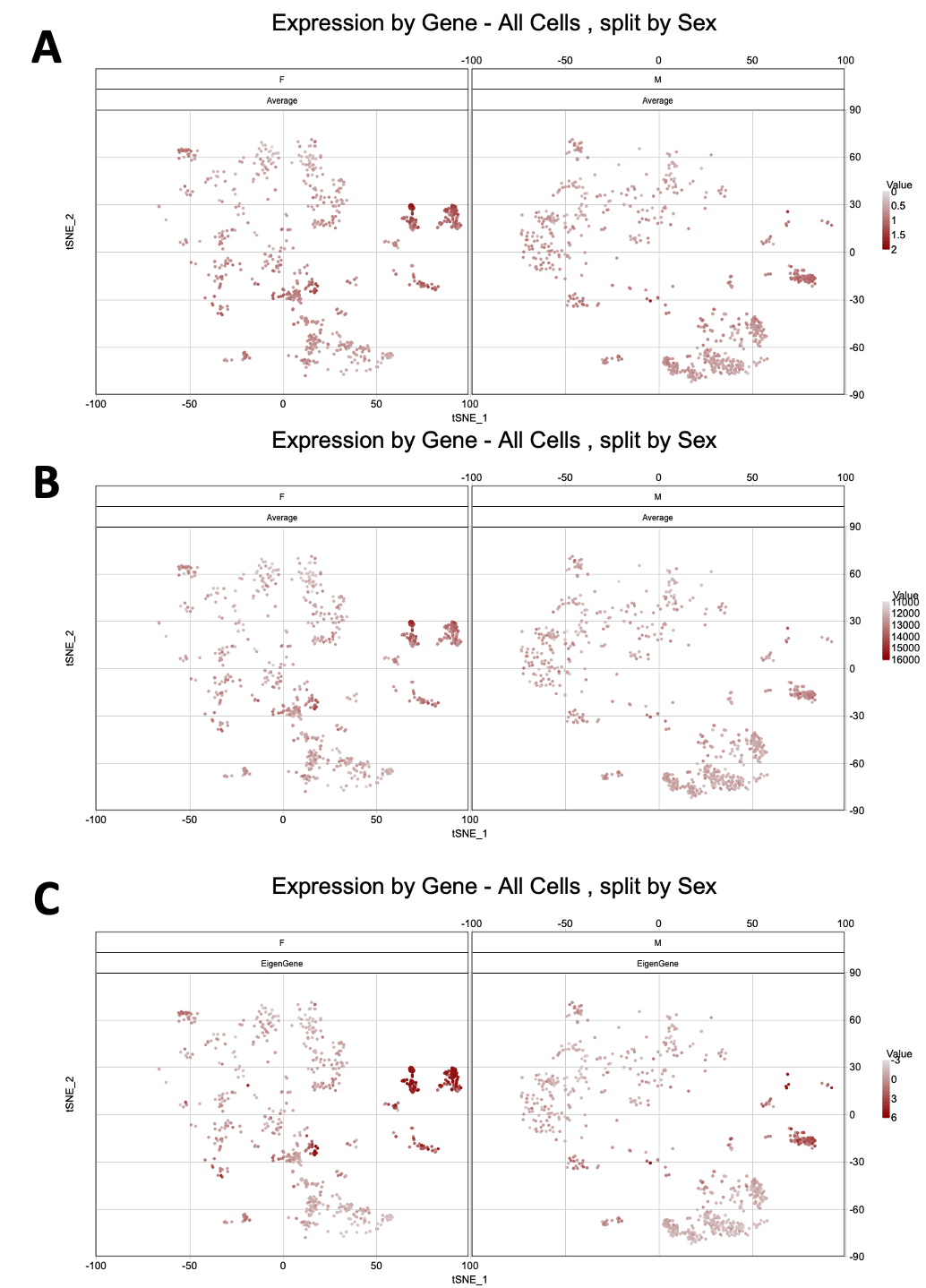


**Figure S2. Sex disparity in activeness of fibroblast growth factor binding.** The disparity is found in GO: fibroblast growth factor binding function activeness inferred by (A) average expression, (B) average rank and (C) eigen-gene expression. Darker color indicates higher activity. The cell clusters with darker color in the F group are cancer-associated fibroblasts and Endothelial cells. F: Female, M: Male


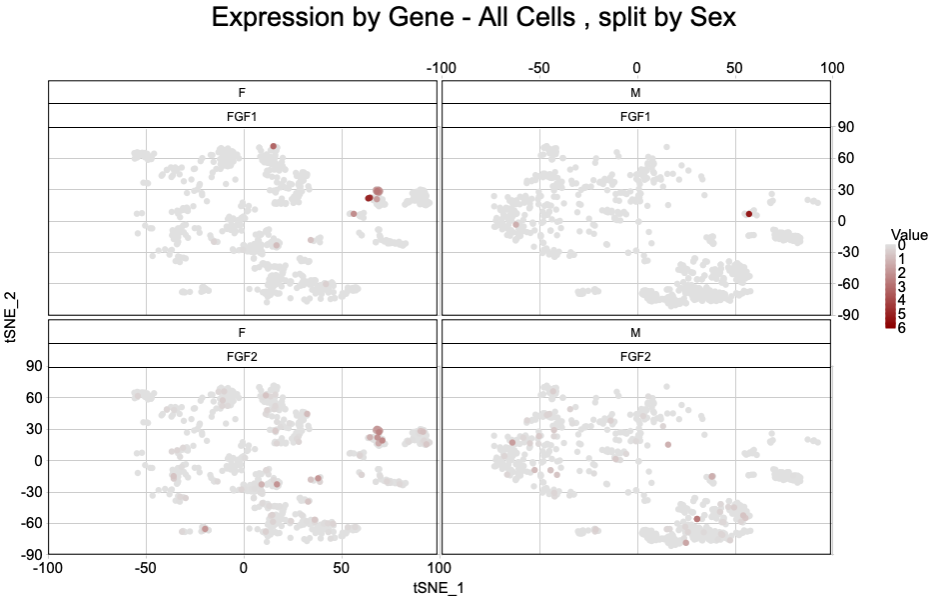


Figure S3. Sex disparity in expression of *FGF1* and *FGF2* expression. Darker color indicates higher expression. The cell clusters with darker color in the F group are cancer-associated fibroblasts and Endothelial cells. F: Female, M: Male.


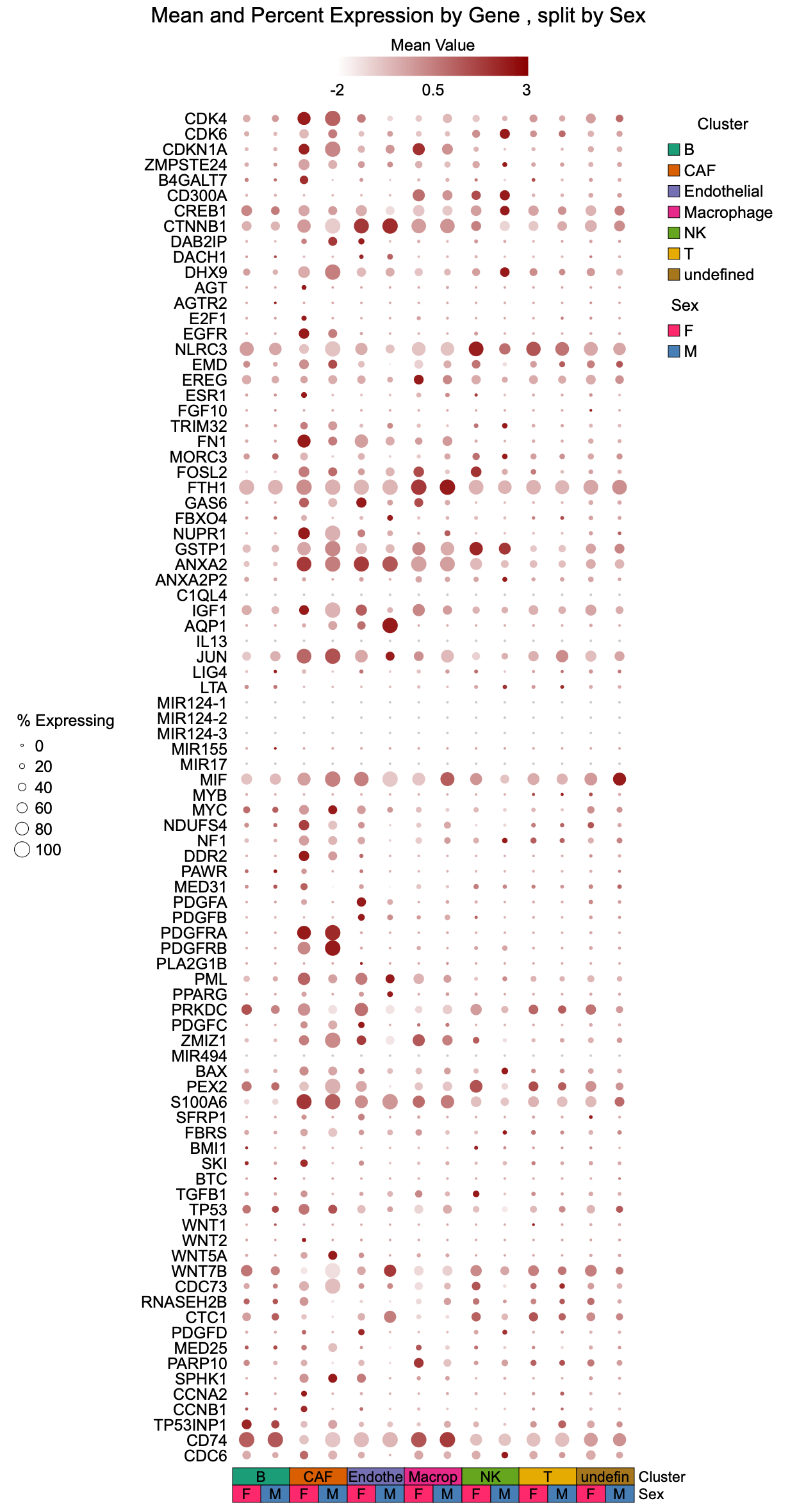


Figure S4. Sex disparity in expression of fibroblast growth factor binding related genes. Darker color indicates higher expression. Larger size indicates higher detection rate. F: Female, M: Male.

**
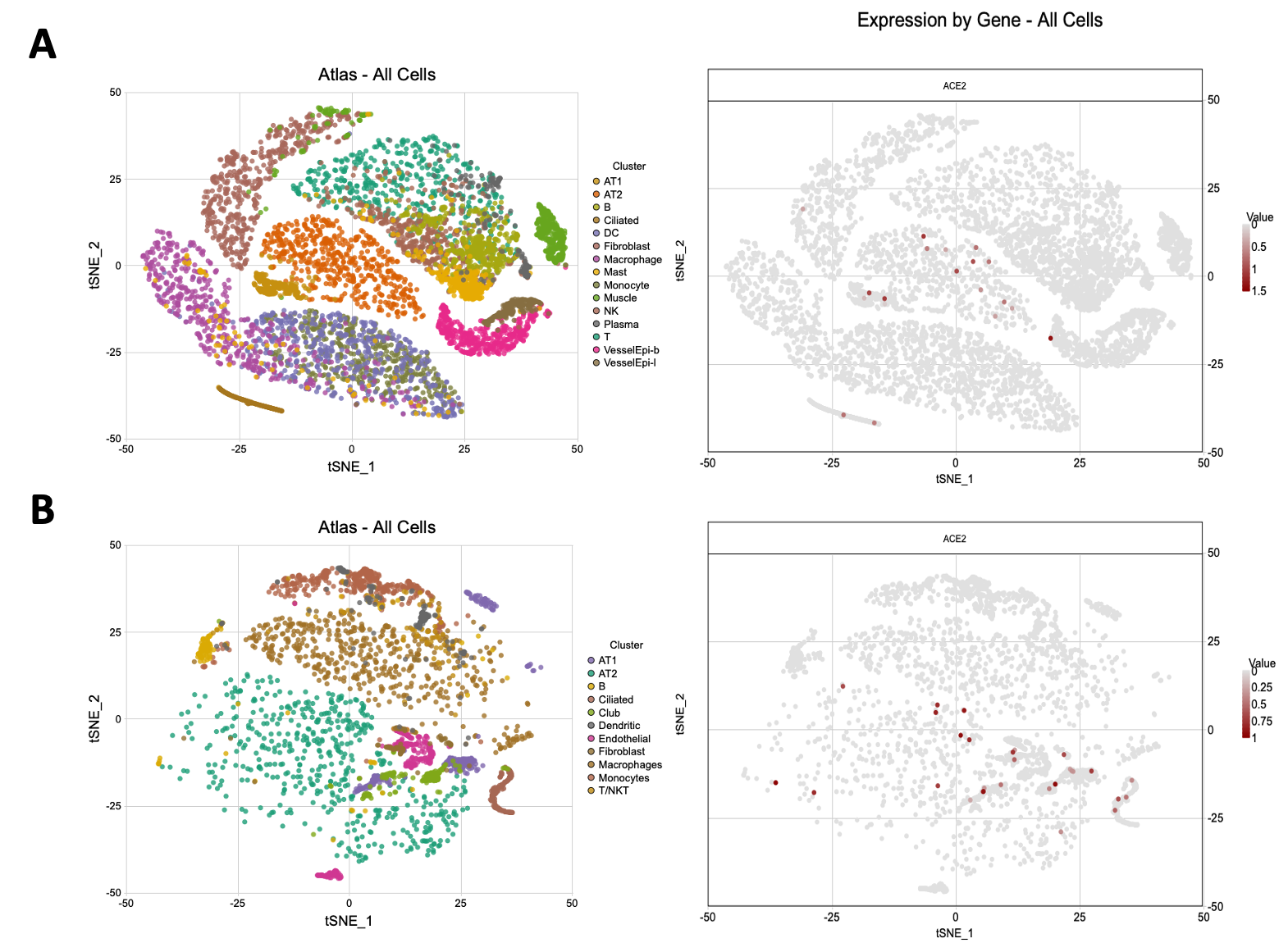
**

**Figure S5. SCANNER detected *ACE2* expression in specific lung cells.** Two datasets including (A) the GSE122960 dataset and (B) the Meyer dataset shows *ACE* expression (right panel) mainly expressed in pneumocytes, secretory cells and ciliated cells (left panel).
